# *rac*-Carbon­yl{1-[(diphenyl­phosphino)meth­yl]ethanethiol­ato}(triphenyl­phosphine)rhodium(I)

**DOI:** 10.1107/S1600536808034284

**Published:** 2008-10-25

**Authors:** Simón Hernández-Ortega, David Morales-Morales

**Affiliations:** a Instituto de Química, Universidad Nacional Autónoma de México, Circuito Exterior, Ciudad Universitaria, México 04510, Mexico

## Abstract

The title compound, [Rh(C_15_H_16_PS)(C_18_H_15_P)(CO)], was synthesized from the reaction of the ligand *rac*-[Ph_2_PCH_2_CH(CH_3_)SH] with *trans*-[Rh(F)(CO)(PPh_3_)_2_] in a 1:1 molar ratio in toluene. The Rh atom is four-coordinated in a distorted square-planar geometry with the P—S ligand [Ph_2_PCH_2_CH(CH_3_)S] acting as a chelate and the PPh_3_ and disordered CO [site occupation factors of 0.61 (5) and 0.39 (5)] ligands completing the coordination.

## Related literature

For general background, see: Au-Yeung & Chan (2004 ▶); Braunstein & Naud (2001 ▶); Dilworth & Weatley (2000 ▶); Dilworth *et al.* (2000 ▶); Fierro-Arias *et al.* (2008 ▶); Gómez-Benítez *et al.* (2007 ▶); Morales-Morales *et al.* (2002 ▶); Xie & Zhou (2008 ▶). For related structures, see: Lee *et al.* (2002 ▶).
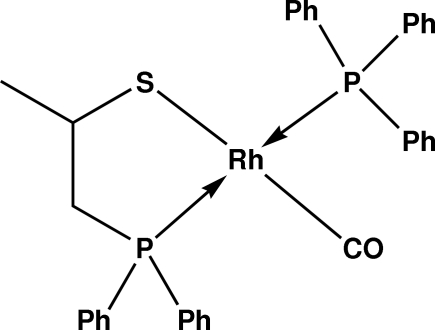


## Experimental

### 

#### Crystal data

[Rh(C_15_H_16_PS)(C_18_H_15_P)(CO)]
*M* *_r_* = 652.50
Orthorhombic, 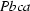

*a* = 10.3142 (7) Å
*b* = 16.865 (1) Å
*c* = 34.984 (2) Å
*V* = 6085.5 (6) Å^3^
*Z* = 8
Mo *K*α radiation
μ = 0.76 mm^−1^
*T* = 298 (2) K
0.26 × 0.23 × 0.03 mm

#### Data collection

Bruker SMART APEX CCD area-detector diffractometer
Absorption correction: multi-scan (*SADABS*; Sheldrick, 1996 ▶) *T*_min_ = 0.827, *T*_max_ = 0.978
48521 measured reflections
5573 independent reflections
4152 reflections with *I* > 2σ(*I*)
*R*_int_ = 0.095

#### Refinement

*R*[*F* ^2^ > 2σ(*F* ^2^)] = 0.049
*wR*(*F* ^2^) = 0.108
*S* = 1.06
5573 reflections
372 parameters
45 restraints
H-atom parameters constrained
Δρ_max_ = 0.65 e Å^−3^
Δρ_min_ = −0.49 e Å^−3^

### 

Data collection: *SMART* (Bruker, 1999 ▶); cell refinement: *SAINT-Plus* (Bruker, 1999 ▶); data reduction: *SAINT-Plus*; program(s) used to solve structure: *SHELXTL* (Sheldrick, 2008 ▶); program(s) used to refine structure: *SHELXTL*; molecular graphics: *ORTEPIII* (Burnett & Johnson, 1996 ▶) and *ORTEP-3 for Windows* (Farrugia, 1997 ▶); software used to prepare material for publication: *SHELXTL*.

## Supplementary Material

Crystal structure: contains datablocks I, global. DOI: 10.1107/S1600536808034284/dn2386sup1.cif

Structure factors: contains datablocks I. DOI: 10.1107/S1600536808034284/dn2386Isup2.hkl

Additional supplementary materials:  crystallographic information; 3D view; checkCIF report

## Figures and Tables

**Fig. 1.**
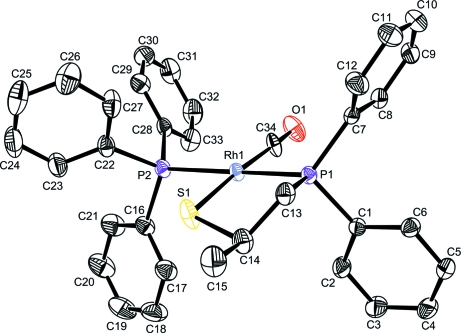
Molecular structure of (I) with the atom-labeling scheme. Displacement ellipsoids are drawn at the 30% probability level. Disordered atom and hydrogen atoms were omitted for clarity.

## supplementary crystallographic information

### Comment

In recent years, attention has increasingly been paid to the coordination chemistry of polydentate ligands incorporating both thiolate and tertiary phosphine donor ligands, as their combination is likely to confer unusual structures and reactivities on their metal complexes [Dilworth, *et al.* 2000, Morales-Morales, *et al.*, 2002]. In the specific case of compounds with platinum group metals these may be suitable species for catalytic screening. Addiconally, the presence of these ligands in their transition metal complexes may render interesting behaviors in solution as these ligands can be capable of full or partial deligation (hemilability), (Dilworth & Weatley, 2000, Braunstein & Naud, 2001) being able to provide important extra coordination sites for incoming substrates during a catalytic process ([Dilworth & Weatley, 2000, Braunstein & Naud, 2001). Moreover, chiral or potentially bidentate ligands have been used extensively to perform asymmetric transformations [Au-Yeung, *et al.* 2004, & Xie *et al.*, 2008], however the most commonly employed are bidentated phosphines and the use of sulfur containing ligands has been avoided owing to the well known propensity of platinum group metals to sulfur poisoning.

Thus, owing to our continuous interest in the synthesis of transition metal complexes bearing P—S hybrid ligands [Morales-Morales, *et al.*, 2002, Gómez-Benítez, *et al.*, 2007, Fierro-Arias, *et al.* 2008] we would like to report the crystal structure of the rhodium(I) complex [Rh(Ph_2_PCH_2_CH(CH_3_)S)(PPh_3_)(CO)] (I).

The rhodium atom is four-coordinated in a distorted square planar geometry with the P—S ligand [Ph_2_PCH_2_CH(CH_3_)S] acting as a chelate and the PPh_3_ and CO ligands completing the coordination sphere (Fig. 1). Similar geometry has been found in a previously reported rhodium complex (Lee *et al.*, 2002). The phenyl ring on the P atoms are essentially planar, these phenyl rings are rotated around the P—C bond, forming the dihedral angles with the coordination plane P1-C34-P2-S1 (Table 1).

### Experimental

Synthesis of [Rh(Ph_2_PCH_2_CH(CH_3_)S)(PPh_3_)(CO)] (1). To a solution of *trans*-[Rh(F)(CO)(PPh_3_)_2_] (100 mg, 12 mmol) in toluene (25 ml) 1 equivalent of the ligand rac-[Ph_2_PCH_2_CH(CH_3_)SH] in toluene (10 ml) was added under stirring. The resulting mixture was allowed to stir overnight. After this time, the solvent was taken off under vacuum and the residue recrystallized from a double layer solvent system CH_2_Cl_2_/MeOH to afford complex 1 as a microcrystalline yellow powder. Yield 87%. ^1^H-NMR (300 MHz, CDCl_3_), (7.00–8.00 (m, Ph, 25H), 2.90–3.20 (m, CH_2_, 2H), 2.40–2.70 (m, CH, 1H), 1.30–1.50 (d, CH_3_, 3H); ^31^P-NMR (121 MHz, CDCl_3_), (68.21 (dd), 59.86 (dd) 1JRh-P= 133.4 Hz, 2JP-P = 304.2 Hz. Elem. Anal. Calculated for [C_34_H_31_OP_2_RhS] Calc. %: C: 62.49, H: 4.94. Found %: C: 62.50, H: 4.90. MS-FAB+ [*M*+] = 653 m/*z*.

### Refinement

All H atoms were fixed geometrically and treated as riding with C—H = 0.98 Å (methyne), 0.97 Å (methylene), 0.96Å (methyl) and 0.93Å (aromatic) with U_iso_(H)= 1.2U_eq_(aroatic, methylene, methine) or U_iso_(H) = 1.5U_eq_(methyl).

The CO is disordered and was refined anisotropically in two major contributors (61/39% for C34,O1/C34A,O1A, respectively)

### Crystal data

**Table:**

[Rh(C_15_H_16_PS)(C_18_H_15_P)(CO)]	*F*(000) = 2672
*M**_r_* = 652.50	*D*_x_ = 1.424 Mg m^−^^3^
Orthorhombic, *P**b**c**a*	Mo *K*α radiation, λ = 0.71073 Å
Hall symbol: -P 2ac 2ab	Cell parameters from 7386 reflections
*a* = 10.3142 (7) Å	θ = 2.3–31.0°
*b* = 16.865 (1) Å	µ = 0.76 mm^−^^1^
*c* = 34.984 (2) Å	*T* = 298 K
*V* = 6085.5 (6) Å^3^	Prism, yellow
*Z* = 8	0.26 × 0.23 × 0.03 mm

### Data collection

**Table:**

Bruker SMART APEX CCD area-detector diffractometer	5573 independent reflections
Radiation source: fine-focus sealed tube	4152 reflections with *I* > 2σ(*I*)
graphite	*R*_int_ = 0.095
Detector resolution: 0.83 pixels mm^-1^	θ_max_ = 25.4°, θ_min_ = 2.3°
ω scans	*h* = −12→12
Absorption correction: multi-scan (SADABS; Sheldrick, 1996)	*k* = −20→20
*T*_min_ = 0.827, *T*_max_ = 0.978	*l* = −42→42
48521 measured reflections	

### Refinement

**Table:**

Refinement on *F*^2^	Primary atom site location: structure-invariant direct methods
Least-squares matrix: full	Secondary atom site location: difference Fourier map
*R*[*F*^2^ > 2σ(*F*^2^)] = 0.049	Hydrogen site location: inferred from neighbouring sites
*wR*(*F*^2^) = 0.108	H-atom parameters constrained
*S* = 1.06	*w* = 1/[σ^2^(*F*_o_^2^) + (0.0403*P*)^2^ + 6.156*P*] where *P* = (*F*_o_^2^ + 2*F*_c_^2^)/3
5573 reflections	(Δ/σ)_max_ = 0.001
372 parameters	Δρ_max_ = 0.65 e Å^−^^3^
45 restraints	Δρ_min_ = −0.49 e Å^−^^3^

### Special details

**Table:**

Geometry. All e.s.d.'s (except the e.s.d. in the dihedral angle between two l.s. planes) are estimated using the full covariance matrix. The cell e.s.d.'s are taken into account individually in the estimation of e.s.d.'s in distances, angles and torsion angles; correlations between e.s.d.'s in cell parameters are only used when they are defined by crystal symmetry. An approximate (isotropic) treatment of cell e.s.d.'s is used for estimating e.s.d.'s involving l.s. planes.
Refinement. Refinement of *F*^2^ against ALL reflections. The weighted *R*-factor *wR* and goodness of fit *S* are based on *F*^2^, conventional *R*-factors *R* are based on *F*, with *F* set to zero for negative *F*^2^. The threshold expression of *F*^2^ > σ(*F*^2^) is used only for calculating *R*-factors(gt) *etc*. and is not relevant to the choice of reflections for refinement. *R*-factors based on *F*^2^ are statistically about twice as large as those based on *F*, and *R*- factors based on ALL data will be even larger.

### Fractional atomic coordinates and isotropic or equivalent isotropic displacement parameters (Å^2^)

**Table:**

	*x*	*y*	*z*	*U*_iso_*/*U*_eq_	Occ. (<1)
Rh1	0.32255 (3)	0.37305 (2)	0.105932 (9)	0.03228 (11)	
C34	0.1592 (15)	0.377 (2)	0.0860 (10)	0.047 (4)	0.61 (5)
O1	0.0553 (7)	0.3812 (17)	0.0750 (4)	0.067 (4)	0.61 (5)
C34A	0.154 (2)	0.363 (3)	0.0902 (18)	0.050 (5)	0.39 (5)
O1A	0.0544 (11)	0.344 (2)	0.0794 (8)	0.068 (5)	0.39 (5)
S1	0.53111 (12)	0.35920 (9)	0.13006 (4)	0.0596 (4)	
P1	0.40804 (11)	0.32103 (6)	0.05089 (3)	0.0316 (3)	
P2	0.26119 (11)	0.43243 (7)	0.16325 (3)	0.0357 (3)	
C1	0.3715 (4)	0.2171 (2)	0.04190 (12)	0.0331 (9)	
C2	0.3274 (5)	0.1695 (3)	0.07131 (13)	0.0519 (13)	
H2	0.3099	0.1913	0.0952	0.062*	
C3	0.3096 (6)	0.0894 (3)	0.06497 (15)	0.0610 (15)	
H3	0.2819	0.0573	0.0850	0.073*	
C4	0.3320 (5)	0.0561 (3)	0.02980 (15)	0.0555 (14)	
H4	0.3186	0.0021	0.0260	0.067*	
C5	0.3742 (5)	0.1030 (3)	0.00031 (14)	0.0481 (12)	
H5	0.3891	0.0810	−0.0237	0.058*	
C6	0.3945 (4)	0.1826 (3)	0.00618 (12)	0.0398 (10)	
H6	0.4239	0.2140	−0.0139	0.048*	
C7	0.3736 (4)	0.3667 (2)	0.00480 (11)	0.0340 (10)	
C8	0.2469 (5)	0.3649 (3)	−0.00953 (13)	0.0435 (11)	
H8	0.1823	0.3391	0.0042	0.052*	
C9	0.2169 (6)	0.4009 (3)	−0.04367 (14)	0.0540 (13)	
H9	0.1326	0.3987	−0.0530	0.065*	
C10	0.3106 (7)	0.4400 (3)	−0.06404 (15)	0.0661 (16)	
H10	0.2893	0.4650	−0.0869	0.079*	
C11	0.4348 (7)	0.4422 (3)	−0.05088 (15)	0.0677 (17)	
H11	0.4983	0.4682	−0.0650	0.081*	
C12	0.4671 (5)	0.4061 (3)	−0.01658 (14)	0.0542 (13)	
H12	0.5521	0.4082	−0.0078	0.065*	
C13	0.5832 (4)	0.3222 (3)	0.05573 (12)	0.0400 (11)	
H13A	0.6223	0.2877	0.0368	0.048*	
H13B	0.6159	0.3755	0.0519	0.048*	
C14	0.6172 (5)	0.2934 (3)	0.09580 (13)	0.0489 (12)	
H14	0.5864	0.2389	0.0991	0.059*	
C15	0.7617 (5)	0.2968 (4)	0.10406 (16)	0.0695 (17)	
H15A	0.8064	0.2603	0.0876	0.104*	
H15B	0.7770	0.2827	0.1303	0.104*	
H15C	0.7930	0.3496	0.0995	0.104*	
C16	0.2358 (5)	0.3652 (3)	0.20339 (13)	0.0448 (12)	
C17	0.2788 (5)	0.2880 (3)	0.20110 (15)	0.0578 (14)	
H17	0.3237	0.2708	0.1796	0.069*	
C18	0.2550 (7)	0.2360 (4)	0.23093 (17)	0.0781 (19)	
H18	0.2830	0.1837	0.2293	0.094*	
C19	0.1906 (6)	0.2614 (4)	0.26265 (18)	0.081 (2)	
H19	0.1741	0.2260	0.2824	0.098*	
C20	0.1502 (6)	0.3382 (5)	0.26568 (16)	0.080 (2)	
H20	0.1097	0.3556	0.2879	0.096*	
C21	0.1692 (6)	0.3896 (4)	0.23600 (15)	0.0683 (17)	
H21	0.1376	0.4411	0.2376	0.082*	
C22	0.3760 (4)	0.5072 (3)	0.17966 (13)	0.0432 (11)	
C23	0.4103 (6)	0.5192 (3)	0.21715 (15)	0.0634 (15)	
H23	0.3800	0.4849	0.2359	0.076*	
C24	0.4899 (7)	0.5821 (4)	0.2272 (2)	0.086 (2)	
H24	0.5136	0.5894	0.2526	0.103*	
C25	0.5334 (6)	0.6333 (4)	0.2000 (2)	0.086 (2)	
H25	0.5826	0.6771	0.2070	0.103*	
C26	0.5047 (6)	0.6204 (4)	0.1623 (2)	0.0798 (19)	
H26	0.5375	0.6539	0.1436	0.096*	
C27	0.4273 (5)	0.5577 (3)	0.15254 (15)	0.0602 (15)	
H27	0.4087	0.5489	0.1269	0.072*	
C28	0.1079 (4)	0.4881 (3)	0.16297 (12)	0.0382 (10)	
C29	0.1033 (5)	0.5697 (3)	0.16278 (13)	0.0495 (12)	
H29	0.1800	0.5986	0.1636	0.059*	
C30	−0.0137 (6)	0.6091 (3)	0.16137 (16)	0.0634 (15)	
H30	−0.0152	0.6643	0.1610	0.076*	
C31	−0.1269 (6)	0.5679 (4)	0.16049 (16)	0.0706 (17)	
H31	−0.2052	0.5952	0.1594	0.085*	
C32	−0.1268 (5)	0.4868 (4)	0.16114 (17)	0.0691 (16)	
H32	−0.2043	0.4586	0.1609	0.083*	
C33	−0.0086 (5)	0.4475 (3)	0.16212 (15)	0.0576 (14)	
H33	−0.0077	0.3924	0.1622	0.069*	

### Atomic displacement parameters (Å^2^)

**Table:**

	*U*^11^	*U*^22^	*U*^33^	*U*^12^	*U*^13^	*U*^23^
Rh1	0.03252 (19)	0.0389 (2)	0.02540 (18)	0.00223 (16)	0.00101 (15)	−0.00288 (15)
C34	0.042 (4)	0.076 (10)	0.021 (7)	0.001 (5)	0.000 (4)	−0.009 (6)
O1	0.044 (3)	0.106 (11)	0.052 (4)	−0.004 (4)	−0.013 (3)	0.006 (6)
C34A	0.042 (4)	0.079 (12)	0.028 (10)	−0.011 (6)	0.004 (5)	−0.019 (9)
O1A	0.048 (4)	0.097 (12)	0.057 (8)	−0.022 (6)	−0.009 (5)	−0.013 (9)
S1	0.0400 (7)	0.1022 (12)	0.0366 (7)	0.0153 (7)	−0.0063 (6)	−0.0186 (7)
P1	0.0355 (6)	0.0331 (6)	0.0261 (6)	−0.0007 (5)	0.0030 (5)	−0.0019 (5)
P2	0.0355 (6)	0.0446 (7)	0.0270 (6)	0.0050 (5)	0.0021 (5)	−0.0037 (5)
C1	0.034 (2)	0.033 (2)	0.032 (2)	0.0000 (19)	0.0003 (19)	−0.0014 (18)
C2	0.077 (4)	0.044 (3)	0.034 (3)	−0.005 (3)	0.009 (3)	−0.003 (2)
C3	0.090 (4)	0.039 (3)	0.053 (3)	−0.019 (3)	0.007 (3)	0.007 (2)
C4	0.066 (3)	0.036 (3)	0.064 (3)	−0.008 (3)	−0.014 (3)	−0.007 (2)
C5	0.062 (3)	0.038 (3)	0.044 (3)	−0.001 (2)	−0.001 (2)	−0.008 (2)
C6	0.044 (3)	0.036 (2)	0.039 (2)	0.001 (2)	0.007 (2)	−0.001 (2)
C7	0.047 (2)	0.028 (2)	0.028 (2)	0.003 (2)	0.0034 (19)	−0.0047 (18)
C8	0.055 (3)	0.041 (3)	0.035 (3)	−0.002 (2)	−0.001 (2)	−0.006 (2)
C9	0.072 (4)	0.050 (3)	0.039 (3)	0.011 (3)	−0.012 (3)	−0.005 (2)
C10	0.100 (5)	0.061 (4)	0.038 (3)	0.005 (4)	−0.011 (3)	0.006 (3)
C11	0.100 (5)	0.061 (4)	0.041 (3)	−0.015 (3)	0.017 (3)	0.012 (3)
C12	0.060 (3)	0.060 (3)	0.043 (3)	−0.013 (3)	0.002 (3)	0.009 (2)
C13	0.035 (2)	0.047 (3)	0.038 (3)	0.004 (2)	0.006 (2)	−0.008 (2)
C14	0.044 (3)	0.061 (3)	0.042 (3)	0.001 (3)	0.004 (2)	−0.007 (2)
C15	0.050 (3)	0.088 (4)	0.070 (4)	0.012 (3)	−0.004 (3)	−0.017 (3)
C16	0.045 (3)	0.056 (3)	0.034 (2)	0.008 (2)	0.006 (2)	0.003 (2)
C17	0.065 (4)	0.065 (4)	0.044 (3)	0.010 (3)	0.006 (3)	0.005 (3)
C18	0.105 (5)	0.070 (4)	0.059 (4)	0.016 (4)	0.010 (4)	0.024 (3)
C19	0.082 (5)	0.103 (5)	0.059 (4)	−0.001 (4)	0.007 (3)	0.039 (4)
C20	0.083 (5)	0.112 (6)	0.046 (3)	0.017 (4)	0.022 (3)	0.017 (3)
C21	0.082 (4)	0.080 (4)	0.042 (3)	0.016 (3)	0.021 (3)	0.006 (3)
C22	0.034 (2)	0.054 (3)	0.041 (3)	0.005 (2)	−0.004 (2)	−0.011 (2)
C23	0.074 (4)	0.070 (4)	0.046 (3)	0.007 (3)	−0.013 (3)	−0.011 (3)
C24	0.092 (5)	0.100 (5)	0.066 (4)	−0.009 (4)	−0.029 (4)	−0.027 (4)
C25	0.070 (4)	0.094 (5)	0.094 (5)	−0.024 (4)	−0.007 (4)	−0.040 (4)
C26	0.065 (4)	0.088 (5)	0.086 (5)	−0.025 (4)	0.017 (4)	−0.017 (4)
C27	0.056 (3)	0.079 (4)	0.046 (3)	−0.024 (3)	0.003 (3)	−0.014 (3)
C28	0.038 (3)	0.049 (3)	0.027 (2)	0.009 (2)	−0.001 (2)	−0.007 (2)
C29	0.050 (3)	0.058 (3)	0.041 (3)	0.011 (3)	0.006 (2)	−0.001 (2)
C30	0.076 (4)	0.055 (3)	0.059 (3)	0.024 (3)	−0.004 (3)	−0.008 (3)
C31	0.053 (4)	0.095 (5)	0.064 (4)	0.031 (4)	−0.004 (3)	−0.022 (3)
C32	0.041 (3)	0.093 (5)	0.073 (4)	−0.001 (3)	−0.006 (3)	−0.017 (4)
C33	0.043 (3)	0.064 (4)	0.066 (4)	0.001 (3)	0.003 (3)	−0.012 (3)

### Geometric parameters (Å, °)

**Table:**

Rh1—C34	1.824 (7)	C14—C15	1.519 (7)
Rh1—C34A	1.827 (8)	C14—H14	0.9800
Rh1—P1	2.2922 (11)	C15—H15A	0.9600
Rh1—S1	2.3225 (13)	C15—H15B	0.9600
Rh1—P2	2.3289 (11)	C15—H15C	0.9600
C34—O1	1.141 (8)	C16—C17	1.378 (7)
C34A—O1A	1.145 (11)	C16—C21	1.394 (7)
S1—C14	1.859 (5)	C17—C18	1.385 (7)
P1—C13	1.815 (4)	C17—H17	0.9300
P1—C1	1.820 (4)	C18—C19	1.362 (8)
P1—C7	1.822 (4)	C18—H18	0.9300
P2—C22	1.822 (5)	C19—C20	1.365 (9)
P2—C16	1.824 (5)	C19—H19	0.9300
P2—C28	1.840 (4)	C20—C21	1.367 (8)
C1—C2	1.382 (6)	C20—H20	0.9300
C1—C6	1.399 (6)	C21—H21	0.9300
C2—C3	1.382 (7)	C22—C23	1.374 (7)
C2—H2	0.9300	C22—C27	1.381 (7)
C3—C4	1.372 (7)	C23—C24	1.387 (8)
C3—H3	0.9300	C23—H23	0.9300
C4—C5	1.371 (7)	C24—C25	1.362 (9)
C4—H4	0.9300	C24—H24	0.9300
C5—C6	1.374 (6)	C25—C26	1.367 (9)
C5—H5	0.9300	C25—H25	0.9300
C6—H6	0.9300	C26—C27	1.368 (7)
C7—C12	1.390 (6)	C26—H26	0.9300
C7—C8	1.399 (6)	C27—H27	0.9300
C8—C9	1.375 (7)	C28—C29	1.376 (6)
C8—H8	0.9300	C28—C33	1.383 (7)
C9—C10	1.369 (8)	C29—C30	1.379 (7)
C9—H9	0.9300	C29—H29	0.9300
C10—C11	1.362 (8)	C30—C31	1.359 (8)
C10—H10	0.9300	C30—H30	0.9300
C11—C12	1.386 (7)	C31—C32	1.368 (8)
C11—H11	0.9300	C31—H31	0.9300
C12—H12	0.9300	C32—C33	1.388 (7)
C13—C14	1.524 (6)	C32—H32	0.9300
C13—H13A	0.9700	C33—H33	0.9300
C13—H13B	0.9700		
			
C34—Rh1—P1	92.7 (13)	C15—C14—C13	112.9 (4)
C34A—Rh1—P1	94 (2)	C15—C14—S1	108.9 (3)
C34—Rh1—S1	176.0 (12)	C13—C14—S1	107.0 (3)
C34A—Rh1—S1	168.4 (17)	C15—C14—H14	109.3
P1—Rh1—S1	84.87 (4)	C13—C14—H14	109.3
C34—Rh1—P2	93.6 (13)	S1—C14—H14	109.3
C34A—Rh1—P2	92 (2)	C14—C15—H15A	109.5
P1—Rh1—P2	172.88 (4)	C14—C15—H15B	109.5
S1—Rh1—P2	88.97 (4)	H15A—C15—H15B	109.5
O1—C34—Rh1	177 (3)	C14—C15—H15C	109.5
O1A—C34A—Rh1	168 (5)	H15A—C15—H15C	109.5
C14—S1—Rh1	105.56 (16)	H15B—C15—H15C	109.5
C13—P1—C1	103.5 (2)	C17—C16—C21	119.0 (5)
C13—P1—C7	105.8 (2)	C17—C16—P2	119.8 (4)
C1—P1—C7	102.34 (19)	C21—C16—P2	121.2 (4)
C13—P1—Rh1	107.49 (14)	C16—C17—C18	119.8 (5)
C1—P1—Rh1	115.72 (14)	C16—C17—H17	120.1
C7—P1—Rh1	120.42 (14)	C18—C17—H17	120.1
C22—P2—C16	106.3 (2)	C19—C18—C17	120.1 (6)
C22—P2—C28	102.0 (2)	C19—C18—H18	120.0
C16—P2—C28	101.4 (2)	C17—C18—H18	120.0
C22—P2—Rh1	113.09 (16)	C18—C19—C20	120.7 (6)
C16—P2—Rh1	115.75 (16)	C18—C19—H19	119.6
C28—P2—Rh1	116.67 (14)	C20—C19—H19	119.6
C2—C1—C6	118.6 (4)	C19—C20—C21	119.9 (6)
C2—C1—P1	119.9 (3)	C19—C20—H20	120.0
C6—C1—P1	121.3 (3)	C21—C20—H20	120.0
C1—C2—C3	119.5 (4)	C20—C21—C16	120.4 (6)
C1—C2—H2	120.3	C20—C21—H21	119.8
C3—C2—H2	120.3	C16—C21—H21	119.8
C4—C3—C2	121.5 (5)	C23—C22—C27	117.8 (5)
C4—C3—H3	119.3	C23—C22—P2	124.8 (4)
C2—C3—H3	119.3	C27—C22—P2	117.3 (4)
C5—C4—C3	119.4 (5)	C22—C23—C24	120.5 (6)
C5—C4—H4	120.3	C22—C23—H23	119.8
C3—C4—H4	120.3	C24—C23—H23	119.8
C4—C5—C6	120.0 (5)	C25—C24—C23	120.2 (6)
C4—C5—H5	120.0	C25—C24—H24	119.9
C6—C5—H5	120.0	C23—C24—H24	119.9
C5—C6—C1	121.0 (4)	C24—C25—C26	120.1 (6)
C5—C6—H6	119.5	C24—C25—H25	119.9
C1—C6—H6	119.5	C26—C25—H25	119.9
C12—C7—C8	117.7 (4)	C25—C26—C27	119.4 (6)
C12—C7—P1	122.9 (4)	C25—C26—H26	120.3
C8—C7—P1	119.4 (3)	C27—C26—H26	120.3
C9—C8—C7	120.8 (5)	C26—C27—C22	121.9 (5)
C9—C8—H8	119.6	C26—C27—H27	119.1
C7—C8—H8	119.6	C22—C27—H27	119.1
C10—C9—C8	120.4 (5)	C29—C28—C33	117.7 (4)
C10—C9—H9	119.8	C29—C28—P2	122.7 (4)
C8—C9—H9	119.8	C33—C28—P2	119.6 (4)
C11—C10—C9	120.1 (5)	C28—C29—C30	120.8 (5)
C11—C10—H10	120.0	C28—C29—H29	119.6
C9—C10—H10	120.0	C30—C29—H29	119.6
C10—C11—C12	120.4 (5)	C31—C30—C29	120.4 (5)
C10—C11—H11	119.8	C31—C30—H30	119.8
C12—C11—H11	119.8	C29—C30—H30	119.8
C11—C12—C7	120.6 (5)	C30—C31—C32	120.7 (5)
C11—C12—H12	119.7	C30—C31—H31	119.7
C7—C12—H12	119.7	C32—C31—H31	119.7
C14—C13—P1	108.1 (3)	C31—C32—C33	118.6 (6)
C14—C13—H13A	110.1	C31—C32—H32	120.7
P1—C13—H13A	110.1	C33—C32—H32	120.7
C14—C13—H13B	110.1	C28—C33—C32	121.8 (5)
P1—C13—H13B	110.1	C28—C33—H33	119.1
H13A—C13—H13B	108.4	C32—C33—H33	119.1

### Table 1 Dihedral angle of phenyl rings with the coordination center (P1-S1-P2-C34) (Å).

**Table:**

Plane	angle
C1-C6	85.4 (1)
C7-C12	56.29 (9)
C16-C21	74.2 (2)
C22-C27	70.4 (1)
C28-C33	68.9 (1)
